# Character strengths and fluid intelligence

**DOI:** 10.1111/jopy.12715

**Published:** 2022-04-08

**Authors:** André Kretzschmar, Lisa Wagner, Fabian Gander, Jennifer Hofmann, René T. Proyer, Willibald Ruch

**Affiliations:** ^1^ Department of Psychology University of Zurich Zurich Switzerland; ^2^ Jacobs Center for Productive Youth Development University of Zurich Zurich Switzerland; ^3^ Department of Psychology University of Basel Basel Switzerland; ^4^ Department of Applied Psychology University of Applied Sciences Zurich Zurich Switzerland; ^5^ Department of Psychology Martin‐Luther University Halle‐Wittenberg Halle Germany

**Keywords:** character strengths, cognitive ability, validity, VIA‐IS, VIA‐Youth

## Abstract

**Objective:**

Research on the associations between cognitive and noncognitive personality traits has widely neglected character strengths, that means positively and morally valued personality traits that constitute good character.

**Method:**

The present study aimed to bridge this gap by studying the associations between character strengths and fluid intelligence using different operationalizations of character strengths (including self‐ and informant‐reports) and fluid intelligence in children, adolescents, and adults.

**Results:**

The results, based on four samples (*N* = 193/290/330/324), suggested that morally valued personality traits are independent of fluid intelligence, with the exception of love of learning, which showed small but robust positive relationships with fluid intelligence across all samples.

**Conclusions:**

Nonetheless, we argue for further research on the associations with other cognitive abilities and interactions between character strengths and intelligence when examining their relationships with external criteria.

## 1 CHARACTER STRENGTHS AND INTELLIGENCE

The association between noncognitive and cognitive personality traits has been a long‐standing research topic from a theoretical and practical perspective (for an overview, see, e.g., Ackerman & Heggestad, 1997). However, previous research focused heavily on noncognitive personality traits related to the five‐factor model (FFM; Goldberg, 1993) or extensions of this model (e.g., Lee & Ashton, 2004; Musek, 2007). Other noncognitive personality traits have been rather neglected, although it has been repeatedly argued that research should look beyond the FFM of personality (e.g., Ackerman, 2018). As already suggested by Thorndike (1940), especially positive and desirable personality traits might be associated with higher levels of intelligence:

“With few or no exceptions superiority in one desirable trait implies superiority in any other. The various sorts of intelligence … are positively related; *intelligence in general is correlated with virtue and goodwill toward men* [emphasis added]; … all these are correlated with health, poise, sanity, and sensitiveness to beauty. Some of these intercorrelations are low, but they are rarely zero or negative.” (Thorndike, 1940, pp. 273–274)

Character strengths represent such positive and desirable personality traits. Peterson and Seligman (2004) describe character strengths as a family of positively and morally valued traits that together constitute good character. They developed the Values in Action (VIA) classification that encompasses 24 character strengths, which are tentatively summarized under six core virtues^1^: (1) creativity, curiosity, judgment, love of learning, and perspective (assigned to the virtue of wisdom and knowledge); (2) bravery, perseverance, honesty, and zest (virtue of courage); (3) love, kindness, and social intelligence (virtue of humanity); (4) teamwork, fairness, and leadership (virtue of justice); (5) forgiveness, humility, prudence, and self‐regulation (virtue of temperance); and (6) appreciation of beauty and excellence, gratitude, hope, humor, and spirituality (virtue of transcendence).

Character strengths are a part of personality and represent a selected subset of personality traits. Although the FFM provides an overall description of a person, character strengths are narrower, specific traits describing the “good person” (McGrath et al., 2020). Accordingly, a considerable overlap with the personality traits of the FFM is to be expected, but empirical studies have shown that character strengths and FFM personality traits are not redundant (McGrath et al., 2020; Noftle et al., 2011; Ruch et al., 2021). Furthermore, character strengths incrementally explain variance in various work, educational, and behavioral outcomes beyond FFM personality traits (e.g., Harzer et al., 2021; Ruch et al., 2017; Wagner & Ruch, 2021). At the same time, the relationship between character strengths and cognitive personality traits has not been examined thus far. We argue that providing an empirical account of these relations is important, for several reasons. When studying the predictive validity of personality traits, it is important to understand the potential overlaps with other constructs; for example, given a relationship between a trait and an outcome, one might be interested in whether this association can be explained by the shared variance with other constructs, such as cognitive ability. Furthermore, knowledge on the relationships between character strengths and cognitive ability might also yield relevant information for the advancement of theoretical ideas and applications related to character strengths. For example, specific character strengths, such as love of learning and curiosity, can be considered “investment personality traits” (i.e., “stable individual differences in the tendency to seek out, engage in, enjoy, and continuously pursue opportunities for effortful cognitive activity”; von Stumm et al., 2011, p. 225) that have been shown to predict knowledge acquisition independent of cognitive ability (von Stumm, 2018). From this perspective, character strengths might shape in which domains (fluid) cognitive abilities are invested but could also foster the accumulation of knowledge and skills eventually (see also Ziegler et al. 2012), helping people foster the virtue of *wisdom*. Finally, studying positively valued traits could also extend the ongoing debate on the associations between personality and cognitive ability (e.g., Ackerman, 2018).

When thinking about potential associations between character strengths and cognitive ability, there are different perspectives one might consider. For example, it has been suggested that personality traits and intelligence are not meaningfully related (Eysenck, 1994). Accordingly, in their handbook introducing the VIA classification and the 24 character strengths, Peterson and Seligman (2004) clearly state that they “excluded (…) talents and abilities (e.g., intelligence)” (p. 15) from the classification. They also elaborate that character strengths are different from abilities because abilities “seem more innate, more immutable, and less voluntary than strengths and virtues” (p. 20) and because character strengths are, in contrast to abilities, morally valued, a claim that has also been empirically supported (Stahlmann & Ruch, 2020). Based on this perspective, one might expect that character strengths and (fluid) intelligence do not overlap, even though both fluid intelligence and, for instance, the character strengths assigned to the virtue of wisdom and knowledge, can result in the acquisition of knowledge. In line with this expectation, earlier studies reported negligible changes in the relationships between character strengths and academic outcomes when controlling for intelligence (Wagner & Ruch, 2021). This idea is also corroborated by earlier findings on similar constructs: Stankov (2018) analyzed the relationships between social attitudes and cognitive ability and reported no correlation between morality (resembling the character strengths assigned to the virtues of humanity and justice) and fluid intelligence. Thus, there are several theoretical and empirical reasons not to expect relationships between character strengths and cognitive ability.

Conversely, there is also a perspective that supports the existence of relationships between specific character strengths and cognitive ability. Ackerman (2018) argued recently that the lack of associations between personality and intelligence may also be due to methodological and conceptual issues and proposed—among other things—going beyond traditional conceptualizations of personality (see also Stankov, 2018). Character strengths might represent such an extension, in line with Thorndike's (1940) early suggestion that “intelligence in general is correlated with virtue and goodwill toward men” (p. 273–274). In his study of social attitudes, Stankov (2018) also reported small negative correlations of “nastiness” (which can be considered the opposite of specific character strengths, such as kindness or modesty) and “religiosity” (similar to the character strength of spirituality) with intelligence, thus providing some empirical evidence for Thorndike's (1940) suggestion. Furthermore, Avey et al. (2012) reported small positive associations between the character strengths assigned to the virtue of wisdom and knowledge (e.g., creativity, curiosity, love of learning, judgment, and perspective) and performance in a verbal creativity task. In a study using caregivers' open‐ended descriptions of children aged 3 to 9 years, love of learning was the only character strength that occurred systematically together with descriptions of a child being intelligent; the effect size, however, was small (Park & Peterson, 2006a). These findings provide tentative hints for a positive association between character strengths, in particular those assigned to the virtue of wisdom and knowledge, and intelligence.

### 1.1 The present study

Overall, there have been theoretical considerations and empirical accounts for both perspectives; namely, that character strengths are related to intelligence, and that these two sets of concepts are unrelated. Accordingly, the present study aimed to examine the associations between all 24 character strengths of the VIA classification (Peterson & Seligman, 2004) and fluid intelligence for the first time, on an exploratory basis.

Study 1 was designed to achieve this aim. Additionally, to obtain more robust results, we used two data sets of previously published studies that originally addressed different research questions (i.e., none of these data sets were used to investigate and report the relation between character strengths and intelligence before). Based on these three data sets, our study considers the two most commonly used questionnaires in character strengths research (i.e., VIA‐IS and VIA‐Youth), self‐ and informant‐reports, different age groups (i.e., children, adolescents, and adults), and different operationalizations of fluid intelligence.

The first study considers the self‐reported character strengths of children and adolescents, whereas gifted students were explicitly recruited for the sample, resulting in a wider distribution of fluid intelligence. Furthermore, the most commonly used operationalization of intelligence was applied, namely, figural fluid reasoning, as in Raven's Matrices Test (Horn, 2009). The second study (data from Wagner et al., 2020) is characterized by a construct representative operationalization of fluid intelligence (Lohman & Lakin, 2011) and self‐reported character strengths of adolescents. The third study (data from the *Zurich Strengths Program*, a large research project described for example in Proyer et al., 2013; Buschor et al., 2013) is characterized by a very broad operationalization of fluid intelligence as well as self‐ and informant‐reports of character strengths of adults (two separate samples). As previous research provided evidence that the character strengths questionnaires for adolescents and adults are not equivalent (Kretzschmar et al., 2022) and self‐ and informant‐ratings of character strengths may correlate differently with other constructs (e.g., Buschor et al., 2013), unique insights into the character strengths‐intelligence relations are expected from this study.

In summary, the present study covers a wide range of assessment‐ and age‐related factors that might have an impact on the association between character strengths and fluid intelligence.

## 2 METHODS

### 2.1 Study 1: Children and adolescents

#### 2.1.1 Participants

In total, 217 students from Swiss primary and secondary schools participated in the study. For the present investigation, we only considered those participants who took part in the intelligence assessment resulting in a final sample of *N* = 193. The mean age was 12.22 years (SD = 2.48) ranging from 7 to 18 years. Gender was almost equally distributed (55% female, 45% male).

#### 2.1.2 Materials

##### Character strengths

The German version (Ruch, Platt, et al., 2014) of the Values in Action Inventory of Strengths for Youth (VIA‐Youth; Park & Peterson, 2006b) consists of 198 items (i.e., 7–9 items per strength); approximately one‐third of the items are negatively keyed. The VIA‐Youth uses a 5‐point Likert‐style format (i.e., 1 = *very much unlike me*; 5 = *very much like me*).

##### Intelligence

The German adaption of Cattell's Culture Fair Intelligence Test (CFT 20‐R; Weiß, 2006) was used. The test consists of four subtests (series, classifications, matrices, and topologies) based on figural task contents. A combined test score was calculated, which represents figural fluid intelligence (gf_fig_) according to the Berlin Intelligence Structure (BIS) model (Jäger, 1982; for an English description; see Süß & Beauducel, 2015).

#### 2.1.3 Procedure

The institutional ethics board of the Faculty of Philosophy at the University of Zurich approved the study procedures. Participants were recruited through schools/teachers and through social media. Both students in classrooms (of regular schools and schools specialized in the education of gifted youth) and individual youth were eligible to participate. Participation was voluntarily and all participants provided written consent. A parent or legal guardian additionally provided written permission in the case of students under the age of 14 years. Participants were offered individualized feedback on their character strengths after completion of the study but received no other compensation. Data were collected either in classrooms or in rooms at the University of Zurich, and data collection was overseen by a master's student. Students first completed the intelligence test and then the VIA‐Youth (and additional measures not relevant to the present study). Both the test and the questionnaires were completed in paper/pencil format.

The data reported here partially overlap with those reported in Ruch, Platt, et al. (2014) who investigated the relationship between character strengths and class clown behavior but did not examine relationships with intelligence.

### 2.2 Study 2: Adolescents

#### 2.2.1 Participants

In the original study (Wagner et al., 2020), 301 students from Swiss secondary schools participated. For the present research question, 11 participants were excluded because they had too little experience with the German language or had missing data on all tasks of the intelligence test resulting in a sample size of *N* = 290. The mean age was 14.46 years (SD = 1.06) ranging from 12 to 19 years. Gender was almost equally distributed (52% female, 48% male).

#### 2.2.2 Materials

##### Character strengths

The VIA‐Youth (Ruch, Weber, et al., 2014) as described in Study 1 was used in the present study.

##### Intelligence

The *Prüfsystem für Schul‐ und Bildungsberatung für 6. bis 13*. *Klassen, Revidierte Fassung* (Testing System for Scholastic and Educational Counseling, Grades 6 to 13—revised version; PSB‐R 6–13; Horn et al., 2004) is an intelligence test designed for educational contexts and consists of nine subtests. A classification according to the BIS model (Jäger, 1982; Süß & Beauducel, 2015) revealed that the subtests mainly assess fluid reasoning, and, to a lesser extent also mental speed, fluency, and general knowledge across verbal, numerical, and figural task contents. Preliminary analysis (see Statistical Analysis section) showed no evidence for considering lower‐order constructs of intelligence in the present sample. Therefore, an overall score for fluid intelligence (gf) was used.

#### 2.2.3 Procedure

The institutional ethics board of the Faculty of Philosophy at the University of Zurich approved the study procedures. Participants were recruited through schools and classroom teachers. All participants participated voluntarily and provided written consent. A parent or legal guardian additionally provided written permission in case of students under the age of 14 years. Participants were offered individualized feedback on their character strengths after completion of the study but received no other compensation. Data were collected in classrooms by two master's students. Students first completed the intelligence test in paper/pencil‐format and then completed the VIA‐Youth (and additional measures not relevant to the present study) on computers or tablets provided by the schools.

The data reported here overlap with those reported in Wagner et al. (2020) and Wagner and Ruch (2021; Study 1), who investigated the relationship between character strengths and educational outcomes while controlling for the influence of intelligence but did not report the relationships between character strengths and intelligence.

### 2.3 Study 3: Adults

#### 2.3.1 Participants

In total, 360 adults who participated in a large research project (see e.g., Proyer et al., 2013) were considered for the present study. We excluded 27 participants who had missing data on the character strengths questionnaire or on all intelligence tests, who did not provide their age, or were identified as outliers (see section Statistical Analysis). In addition, we excluded three participants who had extremely low intelligence test scores that indicated invalid test performance (see Statistical Analysis). Therefore, the final size of the self‐rating sample was *N* = 330. The mean age was 42.47 years (SD = 13.10) ranging from 18 to 77 years. The sample consisted of more women (63%) than men (37%).

For a subsample (*N* = 324), an assessment of character strengths was provided by up to two informants. The mean age of the 616 informant raters was 42.47 years (SD = 14.19) ranging from 18 to 81 years. The sample of informants consists of more women (57%) than men. Most informants were close friends (37%), family members (26%), or partners (20%).

#### 2.3.2 Materials

##### Character strengths

The German version (Ruch et al., 2010) of the Values in Action Inventory of Strengths (VIA‐IS; Peterson et al., 2005) was used. The VIA‐IS consists of 240 positively worded items (10 items per strength) using a 5‐point Likert‐style format (i.e., 1 = *very much unlike me*; 5 = *very much like me*).

Additionally, the informant rating form of the VIA‐IS (VIA‐IS Peer; Ruch et al., 2010) was used. It is identical to the VIA‐IS except that all items are rephrased for informant ratings. The same answer format is used only with rephrased categories (e.g., *very much like him/her*).

##### Intelligence

A broad battery of cognitive tasks was administered. In detail, four subtests (verbal analogies, numeric calculations, figural cubes, verbal memory) from the Intelligence Structure Test 2000 R (IST‐2000‐R; Amthauer et al., 2001), Raven's Standard Progressive Matrices (SPM; Horn, 2009), a vocabulary test (WST; Schmidt & Metzler, 1992), and the attention and concentration test d2 (Brickenkamp, 1998) were administered. According to the BIS model (Jäger, 1982; Süß & Beauducel, 2015), the cognitive measures covered fluid reasoning, mental speed, and short‐term memory across three task contents (i.e., verbal, numerical, figural). However, the vocabulary test was used as an operationalization of verbal crystallized intelligence. As the vocabulary test had insufficient psychometric properties in the present sample, showed nonsubstantial or weak correlations with the other cognitive measurements, and tended to bias the results as a too narrow operationalization (see, e.g., Kretzschmar et al., 2016, 2018), we did not further consider the vocabulary tests for the present investigation. Based on the remaining six subtests, a preliminary analysis (see Statistical Analysis section) showed no evidence for considering lower‐order constructs of intelligence in the present sample. Therefore, a combined score for fluid intelligence (gf) was used.

#### 2.3.3 Procedure

Data presented here were collected among individuals who had registered for a positive psychology training program (Proyer et al., 2013, 2016). According to the guidelines of the University of Zurich, no formal ethics approval was required for this study. Newspaper reports and mailing lists were used to recruit a community sample. Participants had to be at least 18 years old and to not currently be undergoing psychotherapeutic or psychopharmacological treatment or studying psychology. After their registration for the study and providing informed consent, participants were invited to the University of Zurich to complete the tests of cognitive ability (and other measures on positive psychological functioning; not reported here) in group testing sessions, which were supervised by research assistants. Character strengths questionnaires in paper/pencil format were mailed to participants. Additionally, participants were asked to select two people from their immediate environment who knew the participants very well and were potentially good judges of their personality. Participants received informant rating questionnaires to be distributed to these two persons who provided information on the participants' character strengths and mailed the completed questionnaires directly back to the researchers.

### 2.4 Statistical analysis

The statistical approach was the same for all three studies. In the first step, participants who had exclusively missing data on the questionnaires or intelligence tests were excluded (see section Participants). Furthermore, outliers were examined following the approach of Leys et al. (2018) using scale sum scores. Three cases of univariate outliers were identified in Study 3 (i.e., values outside the interval formed by the median plus/minus three times the median absolute deviation), but no cases of multivariate outliers were identified that would have substantially affected the findings. The reliability of each scale was estimated based on McDonald's omega for continuous (i.e., intelligence tests) or categorical (i.e., VIA questionnaires) data (see Flora, 2020).

With regard to the intelligence tests, we classified each subtest according to the BIS model (Jäger, 1982; Süß & Beauducel, 2015). Using confirmatory factor analysis, we examined several measurement models differentiating higher‐ and lower‐order constructs of intelligence (e.g., verbal fluid intelligence, numerical fluid intelligence, or mental speed) for Studies 2 and 3. However, the correlations between the subtest were considerably large so that a differentiation was not possible according to the approach of Gignac and Kretzschmar (2017). Therefore, a measurement model with a general factor was examined based on commonly used evaluation criteria (i.e., CFI ≥ 0.95, RMSEA ≤ 0.08, SRMR ≤ 0.10; Schermelleh‐Engel et al., 2003). The unidimensional measurement models fit the data well for Study 1 and 3, but two residual correlations were added for Study 2 to ensure good model fit.

For the VIA questionnaires, no widely accepted measurement model on item level for the VIA questionnaires exists (see, e.g., Ng et al., 2017) and, thus, using sum scores has been the standard procedure in this field. However, sum scores are also based on a measurement model with rather strict constraints: Sum scores can lead to biased results if the underlying measurement model does not fit the data (McNeish & Wolf, 2020). To ensure unbiased results using psychometrically appropriate measurement models on the one hand, but to ensure comparability with previous studies on the other hand, we applied two approaches in the present study. In the first approach, we examined a unidimensional measurement model for each character strength separately using maximum likelihood estimation with robust standard errors (MLR) and the full information maximum likelihood (FIML) procedure to account for missing data. Almost none of the measurement models showed an acceptable model fit (i.e., CFI ≥ 0.95, RMSEA ≤ 0.08, SRMR ≤ 0.10; Schermelleh‐Engel et al., 2003). In the next step, we sequentially added a residual correlation for each measurement model based on the modification indices until an acceptable model fit was achieved. These data‐driven measurement models were used for further analyses. In the second approach, we calculated mean scale scores for the 24 character strengths. With regard to the informant ratings in Study 3, both ratings were averaged into combined informant rating scores as commonly applied in previous character strength research (see, e.g., Buschor et al., 2013).

To examine the association between character strengths and fluid intelligence, we used the local structural‐after‐measurement approach (Rosseel & Loh, 2021). In detail, the previously verified measurement models for intelligence and character strengths were used to estimate the latent correlations with the maximum likelihood estimation with robust standard errors (MLR) and the FIML procedure. In addition, we report Pearson's correlations based on the sum scale scores. As previous research provided evidence for impact of gender and age on character strengths (e.g., Heintz et al., 2019; Heintz & Ruch, 2021) and intelligence (e.g., Hartshorne & Germine, 2015; Lynn & Irwing, 2004), age and gender were controlled for in all analyses regarding the association between fluid intelligence and character strengths. The covariance matrices and the R code for the main analyses are available as Supplemental Online Material, for an inspection of the detailed results (e.g., measurement models).

For all three studies, the sample sizes were not determined by a‐priori power analyses regarding our research questions (Lakens, 2021). As the present study was not preregistered and should be considered as exploratory (de Groot, 2014), we do not present *p*‐values but rather interpret confidence intervals as plausible values of correlations in the population (Cumming, 2013). According to Gignac and Szodorai (2016), we interpret the latent correlations as small (|*r*| ≥ 0.15), medium (|*r*| ≥ 0.25), and large (|*r*| ≥ 0.35) effect sizes.

All analyses were performed with R (Version 4.0.0; R Core Team, 2020) and the R‐packages data.table (Version 1.14.0; Dowle & Srinivasan, 2021), DataExplorer (Version 0.8.2; Cui, 2020), dplyr (Version 1.0.5; Wickham et al., 2021), lavaan (Version 0.6.8; Rosseel, 2012), MBESS (Version 4.8.0; Kelley, 2020), psych (Version 2.1.3; Revelle, 2021), and Routliers (Version 0.0.0.3; Delacre & Klein, 2019).

## 3 RESULTS

**FIGURE 1:**
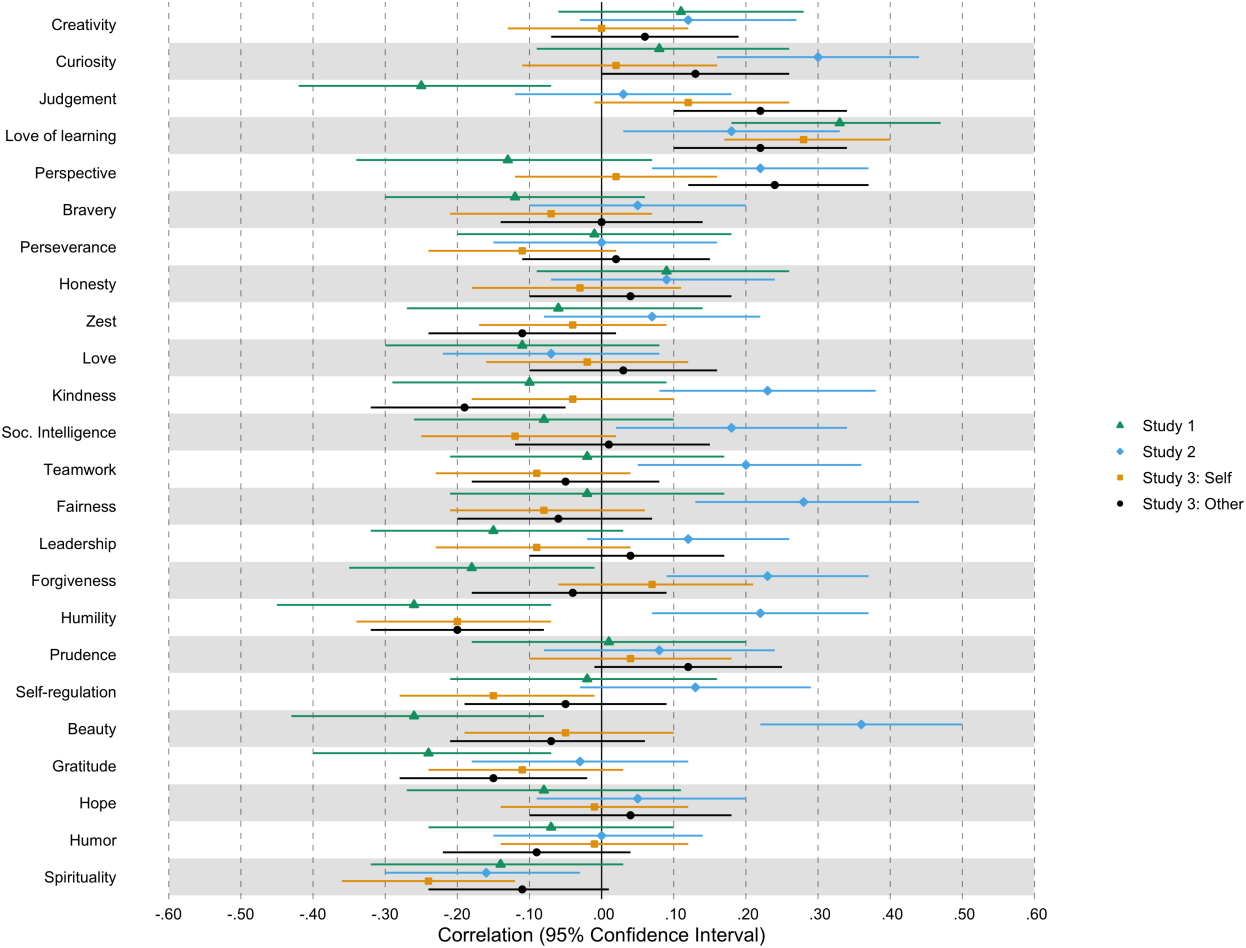
Latent correlations of character strengths and intelligence. Beauty = appreciation of beauty and excellence; Soc. Intelligence = social intelligence. Correlations and 95% CI are displayed in Supplement Table S1. All correlations are controlled for age and gender

Descriptive statistics and reliability estimations of the measures from all three studies are presented in Table 1. Preliminary analyses showed that there was sufficient variability in fluid intelligence and character strengths scores in all samples. Latent correlations between the 24 character strengths and fluid intelligence are shown in Figure 1 and Supplement Table S1 (for observed correlations, see Supplement Table S2). In total, there were 15 substantial correlations (i.e., of at least small effect size; *r*_max_ = 0.36) in at least one study: curiosity, judgment, love of learning, perspective, kindness, social intelligence, teamwork, fairness, leadership, forgiveness, humility, self‐regulation, appreciation of beauty, gratitude, and spirituality. However, love of learning was the only character strength that showed a substantial association across all four samples. The other correlations between character strengths and fluid intelligence can be considered negligible.

**TABLE 1:** Descriptive statistics and reliability

Scales	Study 1			Study 2			Study 3 self‐rating			Study 3 informant‐rating			
	*M*	SD	ω	*M*	SD	ω	*M*	SD	ω	*M*	SD	ω	ICC
Creativity	3.70	0.62	0.80	3.61	0.63	0.88	3.53	0.60	0.89	3.61	0.51	0.90	0.33
Curiosity	3.68	0.58	0.77	3.54	0.58	0.81	3.95	0.50	0.87	3.97	0.42	0.84	0.32
Judgment	3.50	0.53	0.70	3.51	0.56	0.78	3.74	0.44	0.80	3.78	0.45	0.87	0.27
Love of learning	3.65	0.69	0.80	3.44	0.60	0.76	3.80	0.57	0.88	3.88	0.53	0.85	0.45
Perspective	3.58	0.52	0.66	3.66	0.52	0.75	3.48	0.48	0.84	3.64	0.39	0.80	0.19
Bravery	3.67	0.57	0.75	3.73	0.60	0.83	3.48	0.49	0.77	3.67	0.39	0.76	0.19
Perseverance	3.61	0.59	0.75	3.49	0.62	0.82	3.32	0.55	0.85	3.70	0.50	0.89	0.34
Honesty	3.64	0.67	0.85	3.78	0.58	0.85	3.72	0.40	0.71	3.99	0.36	0.75	0.18
Zest	3.70	0.52	0.67	3.51	0.56	0.77	3.49	0.52	0.77	3.62	0.46	0.83	0.40
Love	3.84	0.62	0.82	4.02	0.64	0.86	3.68	0.53	0.78	3.81	0.44	0.82	0.34
Kindness	3.95	0.61	0.85	4.05	0.58	0.85	3.80	0.44	0.74	3.86	0.40	0.80	0.26
Social intelligence	3.59	0.60	0.75	3.76	0.51	0.72	3.59	0.46	0.78	3.66	0.40	0.79	0.22
Teamwork	3.82	0.53	0.72	3.98	0.50	0.76	3.55	0.45	0.72	3.64	0.41	0.82	0.28
Fairness	3.52	0.54	0.64	3.56	0.56	0.75	3.89	0.44	0.79	3.81	0.41	0.84	0.19
Leadership	3.35	0.64	0.79	3.34	0.67	0.86	3.53	0.45	0.79	3.61	0.42	0.85	0.20
Forgiveness	3.80	0.62	0.73	3.74	0.65	0.83	3.51	0.55	0.87	3.53	0.42	0.82	0.18
Humility	3.50	0.48	0.53	3.67	0.57	0.75	3.19	0.53	0.81	3.40	0.53	0.85	0.41
Prudence	3.31	0.56	0.64	3.31	0.59	0.74	3.28	0.49	0.72	3.54	0.45	0.80	0.32
Self‐regulation	3.43	0.60	0.74	3.46	0.61	0.78	3.19	0.54	0.74	3.42	0.51	0.83	0.40
Beauty	3.75	0.74	0.86	3.49	0.70	0.88	3.54	0.50	0.81	3.45	0.49	0.82	0.30
Gratitude	4.05	0.56	0.79	4.17	0.54	0.81	3.68	0.50	0.87	3.58	0.42	0.84	0.24
Hope	3.78	0.54	0.71	3.80	0.60	0.82	3.44	0.56	0.86	3.50	0.47	0.83	0.34
Humor	3.82	0.65	0.82	3.95	0.61	0.82	3.54	0.56	0.87	3.59	0.49	0.88	0.26
Spirituality	3.59	0.98	0.91	3.49	1.01	0.93	2.91	0.81	0.95	3.01	0.67	0.91	0.49
Intelligence	66.26	10.99	0.84	49.26	9.47	0.82	60.83	13.15	0.79	–	–	–	–

Note

Beauty = Appreciation of Beauty. ω = McDonald's Omega. ICC = intraclass correlation coefficient ICC(1,1) between the two informant ratings. Intelligence scores are presented as percent of maximum possible (POMP) scores (Cohen et al., 1999).

## 4 DISCUSSION

Research investigating the relationship between noncognitive and cognitive personality traits often focused on noncognitive personality traits related to the FFM (Goldberg, 1993). The present study provided novel insights into personality‐intelligence relations by considering character strengths as morally valued personality traits (Peterson & Seligman, 2004) for children, adolescents, and adults. Based on previous findings (Kretzschmar et al., 2022), however, it should be kept in mind that a direct comparison of character strengths between children/adolescents and adults is not appropriate, as the age‐specific character strengths questionnaires used in the present study are not equivalent.

With regard to children and adolescents (Study 1 and 2), the substantial associations between character strengths and fluid intelligence cover all virtues. In detail, the character strengths of curiosity, judgement, love of learning, and perspective are considered “cognitive strengths that entail the acquisition and use of knowledge” (Peterson & Seligman, 2004, p. 29) within the virtue of wisdom and knowledge. Therefore, a substantial correlation between these character strengths and fluid intelligence is not surprising. However, creativity as the other character strengths of the virtue of wisdom and knowledge, showed no substantial associations. This finding is rather surprising, as creativity conceptualized as an ability (Guilford, 1967) and the closely related FFM personality trait openness to experiences (e.g., Noftle et al., 2011) usually show consistent and relatively large correlations with intelligence (e.g., Weiss et al., 2021; Ziegler et al., 2012). One possible explanation could be that creativity as a character strength relates more strongly to other aspects of cognitive ability, such as divergent thinking (see e.g., Avey et al., 2012), that was not covered in assessments used in the present study.

The other substantially related character strengths are assigned to humanity (kindness and social intelligence), justice (teamwork, fairness, and leadership), temperance (forgiveness and humility) and transcendence (appreciation of beauty, gratitude, and spirituality). This finding suggests that the personality‐intelligence relations extend to diverse character strengths, and not that only specific character strengths assigned to the virtue of wisdom and knowledge are associated with fluid intelligence. However, we want to emphasize that findings that have not demonstrated robustness across different samples should interpreted cautiously.

Furthermore, numerically smaller associations between character strengths and fluid intelligence were found in Study 1 compared with Study 2. This finding is in line with previous research demonstrating that the breadth and content‐specificity of the intelligence operationalizations substantially influences the intelligence‐personality relations (Kretzschmar et al., 2018). In detail, commonly applied operationalizations representing figural fluid reasoning (e.g., matrices tests) as applied in Study 1 showed significantly weaker associations between cognitive and noncognitive personality traits (e.g., Beauducel et al., 2007; Kretzschmar et al., 2018). Therefore, we conclude that the associations between character strengths and fluid intelligence are most likely underestimated in Study 1.

With regard to adults (Study 3), the character strengths of judgment, love of learning, and perspective (assigned to the virtue wisdom and knowledge) were substantially related to fluid intelligence. Furthermore, the character strengths of kindness (virtue humanity), humility and self‐regulation (virtue temperance) as well as gratitude and spirituality (virtue transcendence) were negatively related to fluid intelligence. While the latter is in line with meta‐analytical findings (Zuckerman et al., 2013), the former might seem surprising as self‐regulation and humility can be expected to go along with better learning as it includes the acknowledgment of one's own limitations and, thus, the need to acquire knowledge (Krumrei‐Mancuso et al., 2020). However, even the related construct of intellectual humility, which is more strongly focused on humility in the intellectual domain has shown inconsistent relationships with cognitive ability (e.g., Krumrei‐Mancuso et al., 2020; Zmigrod et al., 2019). The associations based on the self‐ and the informant‐rating samples were comparable and did not reveal any systematical and substantial differences. Thus, we consider the convergence of the two rating methods as evidence for the robustness of our results.

With regard to the two perspectives presented in the introduction, we interpret our findings as being mostly in line with the notion that character strengths and fluid intelligence are best conceptualized as rather independent. The character strength of love of learning represents an exception as it demonstrated a robust relationship with cognitive ability, in line with what parents observe in their young children (Park & Peterson, 2006a). One possible explanation is that perceived ease in dealing with intellectual content and solving problems (due to higher cognitive ability) promotes a higher preference for engaging in such content, resulting in an increased love of learning. Thus, affective‐motivational traits, such as love of learning, might not only drive the investment of (fluid) cognitive abilities into (crystallized) cognitive resources (in line with ideas of investment trait, e.g., von Stumm, 2018; see also McGrew, 2022), but also represent a consequence of cognitive abilities, as has been suggested for the FFM dimension of openness (Ziegler et al., 2012). However, these and other possible explanations cannot be further explored in cross‐sectional data and remain open for future study.

### 4.1 Limitations and future directions

The findings presented here need to be interpreted in light of some limitations. Even though extensive and established intelligence tests were used, we have only examined a specific, albeit important, part of cognitive abilities, namely fluid intelligence (see, e.g., McGrew, 2009). As crystallized intelligence is more strongly related to noncognitive personality traits than fluid intelligence (Ackerman & Heggestad, 1997), future studies should investigate the association between character strengths and crystallized intelligence. In addition, the hierarchical structure of intelligence (for crystallized intelligence, see, e.g., Schroeders et al., 2021; Steger et al., 2019) should also be taken into account, as previous research has shown that personality‐intelligence relations vary significantly depending on which hierarchical level of cognitive and noncognitive personality traits are being considered (Kretzschmar et al., 2018). Therefore, future studies investigating the correlations between character strengths and intelligence relations will benefit from considering broader operationalizations of intelligence that can also be used to examine abilities with different breadths and contents (see, e.g., Kretzschmar et al., 2017; Kretzschmar & Nebe, 2021).

Furthermore, the character strengths questionnaire for adults used in the present study and most commonly applied in the field was recently revised (McGrath & Wallace, 2019). Since the original and the revised questionnaires are highly comparable (Vylobkova et al., 2022), it is likely that similar relationships can be found based on the revised version, but this would need to be tested empirically and ideally based on larger sample sizes (see Kretzschmar & Gignac, 2019).

With regard to the informant ratings, we applied a simple but commonly used approach in character strength research (see, e.g., Buschor et al., 2013; Ruch et al., 2010), that means averaging the two ratings and analyzing them independent of the self‐rating. The approach of averaging the ratings is based on the assumption that raters are interchangeable (Eid et al., 2008), which is supported by the participant instruction regarding the selection of raters. However, this assumption can be critically questioned with regard to the relatively low to medium intraclass correlations (see Table 1). As the original study design did not take into account any systematic distinction regarding the selection of raters (e.g., parents versus friends), it was not possible to apply more appropriate methods for psychometric modeling self‐ and other‐reports (see, e.g., Lämmle et al., 2021). While this question is outside the scope of the present study, we recommend that future research in the field of character strengths more systematically examines the association between self‐ and informant‐ratings (see, e.g., Vazire, 2010), including, if useful, the data (i.e., covariance matrices) provided as online Supporting Information for the present study.

An important direction for future research could be testing whether and how the interaction between intelligence and character strengths affects outcomes of interest. In line with the perspective of character strengths as investment personality traits (von Stumm, 2018), studying longitudinal trajectories seems to be of particular interest.

## 5 CONCLUSION

The present study extended previous works on the relationships between personality and cognitive ability by studying positively valued traits and their relationship to fluid intelligence in children, adolescents, and adults. The results were in line with earlier findings on the relationship of intelligence to other personality traits and suggested, overall, that character strengths and intelligence were widely unassociated, except for the character strength of love of learning, which showed consistent small positive associations with fluid intelligence across different samples and assessment methods.

## CONFLICT OF INTEREST

Willibald Ruch is a Senior Scientist for the VIA Institute on Character, which holds the copyright to the VIA Inventory of Strengths.

## AUTHOR CONTRIBUTIONS

**André Kretzschmar**: Conceptualization, Formal analysis, Writing—Original Draft, Writing—Review and Editing, Visualization; **Lisa Wagner**: Conceptualization, Investigation, Data Curation, Writing—Original Draft, Writing—Review and Editing; **Fabian Gander**: Conceptualization, Investigation, Data Curation, Writing—Original Draft, Writing—Review and Editing; **Jennifer Hofmann**: Investigation, Writing—Review and Editing; **René T. Proyer**: Conceptualization, Writing—Review and Editing, Supervision, Funding acquisition; **Willibald Ruch**: Conceptualization, Writing—Review and Editing, Supervision, Funding acquisition.

## ETHICS STATEMENT

Data collection was consistent with ethical standards for the treatment of human subjects. Studies 1 and 2 were approved by the institutional ethics board of the Faculty of Philosophy at the University of Zurich. For study 3 (samples 3 and 4), no formal ethics approval was required according to the local university guidelines.

## Supporting information


Table S1‐2

## ENDNOTE

^1^
Please note that the six virtues–24 character strengths classification was not developed as a factor model with a hierarchical structure (Ruch & Stahlmann, 2020). The theoretical association between specific character strengths and the overarching virtues can be manifold, which is in line with mixed empirical results (for an overview, see Wagner & Ruch, in press). As emphasized by Wagner and Ruch (in press), commonly used character strengths questionnaires are suitable to examine the 24 character strengths, but not constructs on a different hierarchical level.
