# Supplementary material for: Character strengths and fluid intelligence
Source: J Pers. 2022 Apr 8;90(6):1057–69. doi: 10.1111/jopy.12715 (PMC9790612; doi:10.1111/jopy.12715)
Supplement: Supplementary file 1 — Table S1‐2 [file JOPY-90-1057-s001.docx]

Online Supplement

The covariance matrices and the R code are available at <https://osf.io/6dw53/>

Supplement Table S1

Latent Correlations of Character Strengths and Intelligence

| Scales | Study 1 | Study 2 | Study 3 Self-Rating | Study 3 Other-Rating |
| --- | --- | --- | --- | --- |
| Creativity | .11 [-.06, .28] | .12 [-.03, .27] | -.00 [-.13, .12] | .06 [-.07, .19] |
| Curiosity | .08 [-.09, .26] | **.30 [ .16, .44]** | .02 [-.11, .16] | .13 [ .00, .26] |
| Judgment | **-.25 [-.42, -.07]** | .03 [-.12, .18] | .12 [-.01, .26] | **.22 [ .10, .34]** |
| Love of learning | **.33 [ .18, .47]** | **.18 [ .03, .33]** | **.28 [ .17, .40]** | **.22 [ .10, .34]** |
| Perspective | -.13 [-.34, .07] | **.22 [ .07, .37]** | .02 [-.12, .16] | **.24 [ .12, .37]** |
| Bravery | -.12 [-.30, .06] | .05 [-.10, .20] | -.07 [-.21, .07] | -.00 [-.14, .14] |
| Perseverance | -.01 [-.20, .18] | .00 [-.15, .16] | -.11 [-.24, .02] | .02 [-.11, .15] |
| Honesty | .09 [-.09, .26] | .09 [-.07, .24] | -.03 [-.18, .11] | .04 [-.10, .18] |
| Zest | -.06 [-.27, .14] | .07 [-.08, .22] | -.04 [-.17, .09] | -.11 [-.24, .02] |
| Love | -.11 [-.30, .08] | -.07 [-.22, .08] | -.02 [-.16, .12] | .03 [-.10, .16] |
| Kindness | -.10 [-.29, .09] | **.23 [ .08, .38]** | -.04 [-.18, .10] | **-.19 [-.32, -.05]** |
| Soc. Intelligence | -.08 [-.26, .10] | **.18 [ .02, .34]** | -.12 [-.25, .02] | .01 [-.12, .15] |
| Teamwork | -.02 [-.21, .17] | **.20 [ .05, .36]** | -.09 [-.23, .04] | -.05 [-.18, .08] |
| Fairness | -.02 [-.21, .17] | **.28 [ .13, .44]** | -.08 [-.21, .06] | -.06 [-.20, .07] |
| Leadership | **-.15 [-.32, .03]** | .12 [-.02, .26] | -.09 [-.23, .04] | .04 [-.10, .17] |
| Forgiveness | **-.18 [-.35, -.01]** | **.23 [ .09, .37]** | .07 [-.06, .21] | -.04 [-.18, .09] |
| Humility | **-.26 [-.45, -.07]** | **.22 [ .07, .37]** | **-.20 [-.34, -.07]** | **-.20 [-.32, -.08]** |
| Prudence | .01 [-.18, .20] | .08 [-.08, .24] | .04 [-.10, .18] | .12 [-.01, .25] |
| Self-regulation | -.02 [-.21, .16] | .13 [-.03, .29] | **-.15 [-.28, -.01]** | -.05 [-.19, .09] |
| Beauty | **-.26 [-.43, -.08]** | **.36 [ .22, .50]** | -.05 [-.19, .10] | -.07 [-.21, .06] |
| Gratitude | **-.24 [-.40, -.07]** | -.03 [-.18, .12] | -.11 [-.24, .03] | **-.15 [-.28, -.02]** |
| Hope | -.08 [-.27, .11] | .05 [-.09, .20] | -.01 [-.14, .12] | .04 [-.10, .18] |
| Humor | -.07 [-.24, .10] | -.00 [-.15, .14] | -.01 [-.14, .12] | -.09 [-.22, .04] |
| Spirituality | -.14 [-.32, .03] | **-.16 [-.30, -.03]** | **-.24 [-.36, -.12]** | -.11 [-.24, .01] |

*Note*. Soc. Intelligence = Social Intelligence, Beauty = Appreciation of Beauty. 95% CI are stated in brackets. All correlations are controlled for age and gender. Latent correlations highlighted in bold represent effect sizes that are interpreted as substantial (i.e., |*r*| ≥ .15) according to Gignac and Szodorai (2016).

Supplement Table S2

Observed Correlations of Character Strengths and Intelligence

| Scales | Study 1 | Study 2 | Study 3 Self-Rating | Study 3 Other-Rating |
| --- | --- | --- | --- | --- |
| Creativity | **.10 [-.04, .24]** | .09 [-.03, .20] | .00 [-.11, .11] | .03 [-.08, .14] |
| Curiosity | **.12 [-.02, .26]** | **.19 [ .07, .29]** | .02 [-.09, .12] | .09 [-.01, .20] |
| Judgment | .00 [-.14, .14] | .07 [-.05, .18] | .07 [-.04, .18] | **.16 [ .05, .26]** |
| Love of learning | **.21 [ .07, .34]** | **.11 [-.00, .23]** | **.21 [ .10, .31]** | **.17 [ .07, .28]** |
| Perspective | .01 [-.13, .16] | **.15 [ .04, .26]** | .05 [-.06, .16] | **.17 [ .06, .27]** |
| Bravery | -.04 [-.18, .11] | .07 [-.05, .18] | -.04 [-.14, .07] | .02 [-.09, .13] |
| Perseverance | .00 [-.14, .15] | -.01 [-.12, .11] | -.05 [-.16, .06] | .04 [-.07, .15] |
| Honesty | .04 [-.10, .18] | .05 [-.07, .16] | -.04 [-.15, .06] | .04 [-.07, .14] |
| Zest | .02 [-.12, .16] | **.10 [-.02, .21]** | -.02 [-.13, .09] | -.08 [-.18, .03] |
| Love | -.05 [-.19, .09] | -.08 [-.19, .04] | .01 [-.09, .12] | .01 [-.10, .11] |
| Kindness | -.06 [-.20, .08] | **.11 [-.01, .22]** | .03 [-.08, .14] | -.07 [-.18, .04] |
| Soc. Intelligence | -.06 [-.20, .08] | .08 [-.04, .19] | -.02 [-.13, .09] | .04 [-.07, .14] |
| Teamwork | -.00 [-.14, .14] | **.14 [ .02, .25]** | -.05 [-.15, .06] | -.04 [-.15, .07] |
| Fairness | .06 [-.08, .20] | **.15 [ .03, .26]** | -.07 [-.17, .04] | -.00 [-.11, .11] |
| Leadership | -.04 [-.18, .10] | .09 [-.03, .20] | -.03 [-.14, .08] | .03 [-.08, .14] |
| Forgiveness | **-.12 [-.25, .02]** | **.18 [ .06, .29]** | .03 [-.08, .14] | -.01 [-.11, .10] |
| Humility | -.08 [-.22, .06] | **.12 [ .01, .24]** | **-.11 [-.22, -.00]** | **-.11 [-.22, -.00]** |
| Prudence | -.00 [-.14, .14] | .05 [-.07, .16] | .01 [-.10, .12] | .09 [-.02, .19] |
| Self-regulation | .04 [-.11, .18] | **.14 [ .02, .25]** | -.09 [-.20, .02] | -.03 [-.14, .08] |
| Beauty | **-.11 [-.25, .03]** | **.20 [ .09, .31]** | -.01 [-.12, .10] | .01 [-.10, .11] |
| Gratitude | -.09 [-.23, .05] | -.04 [-.16, .07] | -.07 [-.18, .04] | -.04 [-.15, .07] |
| Hope | -.01 [-.15, .13] | .05 [-.07, .16] | -.02 [-.13, .08] | .02 [-.09, .13] |
| Humor | .01 [-.13, .15] | -.01 [-.12, .11] | .02 [-.09, .12] | -.03 [-.14, .08] |
| Spirituality | -.08 [-.22, .06] | **-.15 [-.26, -.03]** | **-.15 [-.25, -.04]** | -.07 [-.18, .03] |

*Note*. Soc. Intelligence = Social Intelligence, Beauty = Appreciation of Beauty. 95% CI are stated in brackets. All correlations are controlled for age and gender. Observed correlations highlighted in bold represent effect sizes that are interpreted as substantial (i.e., |r| ≥ .10) according to Gignac and Szodorai (2016).
